# Electrospun nanofiber mats caged the mammalian macrophages on their surfaces and prevented their inflammatory responses independent of the fiber diameter

**DOI:** 10.1038/s41598-024-61450-3

**Published:** 2024-05-29

**Authors:** Furkan Ayaz, Didem Demir, Nimet Bölgen

**Affiliations:** 1 Department of Molecular Biology and Genetics, Faculty of Engineering and Natural Sciences, Biruni University, 34010 Istanbul, Turkey; 2 Department of Chemistry and Chemical Process Technologies, Vocational School of Technical Sciences, Tarsus University, 33343 Tarsus, Mersin, Turkey; 3 Department of Chemical Engineering, Faculty of Engineering, Mersin University, 33343 Mersin, Turkey

**Keywords:** Electrospinning, PCL, Macrophages, Inflammation, Immunotoxicity, Biomaterials, Nanoscale materials

## Abstract

Poly-ε-caprolactone (PCL) has been widely used as biocompatible materials in tissue engineering. They have been used in mammalian cell proliferation to polarization and differentiation. Their modified versions had regulatory activities on mammalian macrophages in vitro. There are also studies suggesting different nanofiber diameters might alter the biological activities of these materials. Based on these cues, we examined the inflammatory activities and adherence properties of mammalian macrophages on electrospun PCL nanofibrous scaffolds formed with PCL having different nanofiber diameters. Our results suggest that macrophages could easily attach and get dispersed on the scaffolds. Macrophages lost their inflammatory cytokine TNF and IL6 production capacity in the presence of LPS when they were incubated on nanofibers. These effects were independent of the mean fiber diameters. Overall, the scaffolds have potential to be used as biocompatible materials to suppress excessive inflammatory reactions during tissue and organ transplantation by caging and suppressing the inflammatory cells.

## Introduction

The general strategy applied in the field of regenerative medicine is to place the cells in scaffolds produced with a tissue engineering approach and then implant them in the patient's body. Tissue engineering scaffolds should be made of biocompatible materials and must provide mechanical support to the transplanted cells until the newly formed or healing tissues are structurally stabilized. In addition, scaffolds should be designed to regulate processes such as proliferation, differentiation and migration of cells^1^. During tissue or organ transfer or implanting foreign materials to support the tissues, the most common problem is immune rejection which starts with inflammatory responses^2–5^. In order to circumvent inflammatory reactions during these processes novel materials should be explored for their biocompatibility as well as immunotoxicity, immunostimulatory and immunomodulatory activities^4,6^. Immunostimulation is activation of immune system cells in the absence of any stimulatory factors such as necrotic cell debris, bacterial or fungal or viral components and metabolic wastes of tumor cells^7^. Having materials with immunostimulatory functions would enable eliciting a desired immune response against a certain disease or most importantly as adjuvant candidate in vaccine formulations^8^. Having immunomodulatory materials would enable regulation of the immune system cells’ reactions to define the type and strength of the immune response^9–11^. PCL has been used extensively as biocompatible material and there are studies focusing on the effect of different types of PCL nanofibers on mammalian cell proliferation, migration, polarization and cellular activities^12–14^. In addition, previous studies have suggested that modified PCL nanofibres may affect macrophage polarisation and function^15,16^ while other studies have indicated that nanofiber diameter may impact the activity of interacting mammalian cells^17^. So far, we have evaluated PCL nanofibrous membranes for various tissue engineering applications, including wound dressing^18^, drug delivery systems^19^, barrier to prevent of post-operative adhesions^20^ and scaffold for reconstruction of cranial bone defects^21^. In all these studies, one of the most important features desired in PCL membranes designed for in vivo systems is that they act for the purpose of application while at the same time not causing any side effects on the immune system. Therefore, in this study, we aimed to reveal the relationship between PCL nanofiber membranes and mammalian macrophages and whether fiber diameter, which is a determining variable in fibrous membranes produced by electrospinning, has any effect on this interaction.

Mammalian macrophages were used in our study since macrophages play a major role in innate immune reactions as first line of defense as well as a bridge between innate and adaptive immunity by modulating the cellular responses^22^. Macrophages produce cytokines to regulate and define the type and strength of the immune response in a tissue^22^. Electrospinning, the method we used to fabricate PCL scaffolds in our studies, is a known and attractive technique to produce fibrous scaffolds for tissue engineering applications by simulating native extracellular matrix of natural tissue with the help of their fibrillary structures. The most important problem encountered with electrospun scaffolds for tissue repair and regeneration is that the small pores defined by densely compressed fibers significantly inhibit cell infiltration and tissue ingrowth^13^. The distance between densely compacted fibers obtained during a conventional electrospinning process can take the form of a planar layer with a distance much smaller than the cell size and the resultant fibrous membrane act as a two-dimensional structure rather than a three-dimensional network. Macrophage activity like other cells can be affected by this topography of electrospun fibrous scaffolds according to the biological scale in micrometric and nanometric range^23^. Macrophages are dominant infiltrating cells of the immune system that respond rapidly to biomaterial implantation in tissues by releasing both proinflammatory cytokines and antimicrobial mediators^24^. They are found to actively respond to almost all medical devices and tissue engineering scaffolds after following the implantation of these foreign materials in the body such as metals, ceramics, cements, polymers and their composites.

Based on all this information, in our study, the interaction of electrospun PCL scaffolds with macrophages and whether the changing topography by changing the average fibre diameter has any determinant effect on macrophage binding behaviour and immunomodulatory activity were investigated. In this context, PCL electrospun mats with three different average fiber diameters obtained by changing the electrospinning process parameters were evaluated.

## Materials and methods

### Materials

PCL with an average molecular weight of 80 kDa was obtained from Sigma-Aldrich, United Kingdom. Chloroform and dimethylformamide were purchased from Tekkim, Turkey and Carlo Erba Reagents, France, respectively . All chemicals were of analytical grade and used as received.

### Experimental design for fabrication of PCL nanofibers using electrospinning

The experimental conditions selected for PCL nanofibrous scaffolds with different fiber diameters were determined by using the experimental sets created by response surface methodology in another study we previously reported^25^. Briefly, a polymer solution was prepared by dissolving the calculated amount of PCL in a solvent mixture containing 30:70 volumetric ratio of chloroform and dimethylformamide, respectively under continuous stirring for at least 120 min. 2 mL of the prepared solution was placed into a 2.5 mL of plastic syringe and and the syringe was placed in a syringe pump (New Era, NE-1002X, USA) to drive the polymer solution at a constant speed. A high-voltage power supply (Gamma High Voltage Research, USA) was connected to the syringe needle tip and to a collector covered with an aluminum foil. The selected voltage was applied for 60 min to produce an electrospun mat. To obtain scaffolds at three different avarage fiber diameters, the experimental parameters including polymer concentration, flow rate, distance from tip to the collector and voltage were varied. The process and solution parameters used for the electrospinning of nanofibers with different fiber diameters and the resultant sample names were shown in Table 1.

**Table 1:** The process and solution parameters used for the electrospinning of PCL nanofibers.

Sample	PCL concentration (% wt)	Voltage (kV)	Distance (cm)	Flow rate (mL/h)
PCL1	11	11	12	1
PCL2	13	13	10	1
PCL3	13	13	8	2

### Fiber size measurement

The morphologies of the obtained samples were analyzed via scanning electron microscopy (SEM; Supra 55, Zeiss, Germany) at an accelerating voltage of 5 kV with different magnifications, and the mean diameter of the fibers were analyzed by using an image visualization software (Image-J).

### Cell culture studies for SEM analysis of macrophage adherence behavior and inflammatory cytokine ELISAs

RAW 264.7 murine macrophage cell line had ATCC origins and were grown in 1640 RPMI media supplemented with 10% FBS and 1% Pen/Strep antibiotics. 37 °C 5% CO_2_ incubator was used for cell growth and incubation. The cells were incubated on 24 well plates with 1 × 10^6^ cells/mL concentration. For negative and positive control groups no nanofibers were used. In positive control groups and stimulated groups, 1 µg/mL of final concentration of LPS was used. LPS was purchased from ENZO and had origins from *E.coli*. PCL1-3 nanofibers were used separately by following the same below procedure. The nanofibers were in a size to cover the bottom of the 24 well plates. Each well was covered with appropriate nanofiber group and the cells were incubated on top of the nanofibers and rested overnight (12 h) before stimulations. After overnight resting 1 µg/mL of LPS was used to activate the macrophages and the cells were incubated 24 h. After 24 h incubation the supernatants were collected for TNFα and IL6 ELISAs by using BD kit and guidance for the protocol as indicated in our previous publications^26–38^.

To determine the cell adhesion/attachment behavior of the macrophages on PCL nanofibers, after removing the supernatants for ELISA, the cells were fixed on fibrous scaffolds. Glutaraldehyde solution (2.5%, v/v) was added in each well to fill the scaffold surface completely. The scaffolds in the glutaraldehyde solution incubated into a refrigerator at 4 °C for 2 h. Then, the samples were taken into sterile PBS before dehydration. Finally, the PBS solution was decanted and dehydration of the scaffolds was performed in series of alcohol solution for 10 min (50, 70, 90 and 100%, v/v). The dehydrated scaffolds were dried in a desiccator at room temperature. After drying, the samples were coated with platinum for SEM (SEM, Supra 55, Zeiss, Germany) analysis. SEM analysis was performed with an operating voltage of 5 kV at different magnifications. All conditions were repeated at least 3 times (N = 3) and t test was conducted by using GraphPad Prism version 5 program for statistical analysis^26–38^.

## Results

PCL is a FDA approved synthetic polymer that is widely studied for soft and hard tissue engineering applications due to its biocompatible nature, degradability, non-toxicity of degradation products and low melting temperature^39,40^. Studies on PCL, which has been among the most preferred polymers in the field of tissue engineering for many years, continue with an increasing trend. Recent studies on scaffolds in different forms produced by combining PCL directly or with different compounds are on materials fabricated by 3D printing^41–43^, and electrospinning^43–45^. In addition to the basic expectations such as biocompatibility, biodegradability and mechanical properties of implantable biomaterials and carrier systems prepared on the basis of this widely used polymer, it is very important to reveal the response to the immune system in terms of the long-term in vivo success of the biomaterial. As examples of studies on this subject, the effects of electrospun PCL fibers loaded with coumarin and/or zinc oxide nanoparticles on the macrophage inflammatory response^46^, PCL nanofiber membranes with aligned and random orientation on macrophage polarization and regulate inflammatory activation^47^, macrophage membrane-functionalized nanofibrous PCL mats and their immunomodulatory effects on macrophage polarization^15^, the immun reponse of chitosan-graft-PCL electrospun membranes^48^ and effect of PCL-amniotic membrane constructed using electrospinning on macrophage polarization and inflammatory microenvironment^49^ were studied. In this context, the difference of our study is to reveal whether the changing fiber diameter, which is one of the most important characteristics of the fibers, will have any effect on the interaction of the widely used PCL electrospun fibers with macrophages and the effect on the immune response when used alone. For this purpose, in the continuation of the study, electrospun PCL membranes with three different fiber diameters in the nano size range, obtained by changing the experimental conditions, were evaluated.

### Morphology of nanofibers

**Figure 1:**
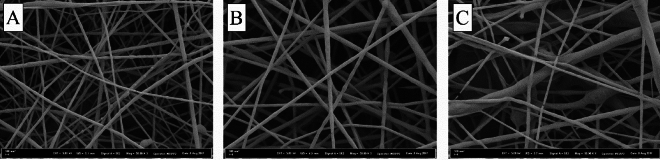
SEM images of nanofibrous scaffolds: (**A**) PCL1, (**B**) PCL2 and (**C**) PCL3.

PCL nanofibers was manufactured using electrospinning technique according to method described in our previous studies (Fig. 1)^19,25^. Various process (tip-to-collector distance, voltage and flow rate) and solution parameters (polymer concentration, viscosity and conductivity) play a role in the formation of fiber structures of different morphologies^50^. In this work, we aimed to show the immunomodulatory activity and macrophage behavior of PCL nanofibers depending on fiber diameter change. Fibrous scaffolds with three different average fiber diameter were produced as a result of the experiments carried out with the parameters in Table 1 given in the experimental section. The mean fiber diameters were calculated as 45.3 ± 7.9, 99.1 ± 15.1 and 140.5 ± 14.1 nm for PCL1, PCL2 and PCL3 scaffolds, respectively. Furthermore, the SEM images of nanofibers are given in Figs. 1A–C, respectively.

### Cell behavior of macrophages on nanofibers

**Figure 2:**
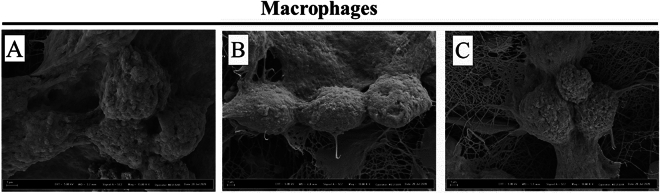
SEM images show the attachment of macrophages on the PCL fibers: (**A**) PCL1; (**B**) PCL2 and (**C**) PCL3.

**Figure 3:**
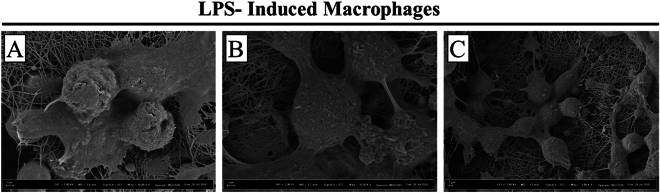
SEM images show the attachment of LPS-induced macrophages on the PCL fibers: (**A**) PCL1; (**B**) PCL2 and (**C**) PCL3.

Previous studies suggest the role of nanofibers to create an environment for mammalian cells to attach and proliferate^51,52^. Macrophages and LPS-induced macrophages were seeded on PCL nanofibrous membranes fabricated at different fiber diameters and results are shown in Fig. 2 and Fig. 3, respectively. SEM images showed that the RAW cells retained their round shape, some of them came together and spread over the surface of the fibers and dispersed widely on the fibrous mat. These sheets created a nest for the macrophages to attach. Due to their dispersed residence on the fibrous mat their functional properties might change which we further deciphered by measuring their inflammatory activities.

### Macrophages lost their inflammatory ability on nanofibers

**Figure 4:**
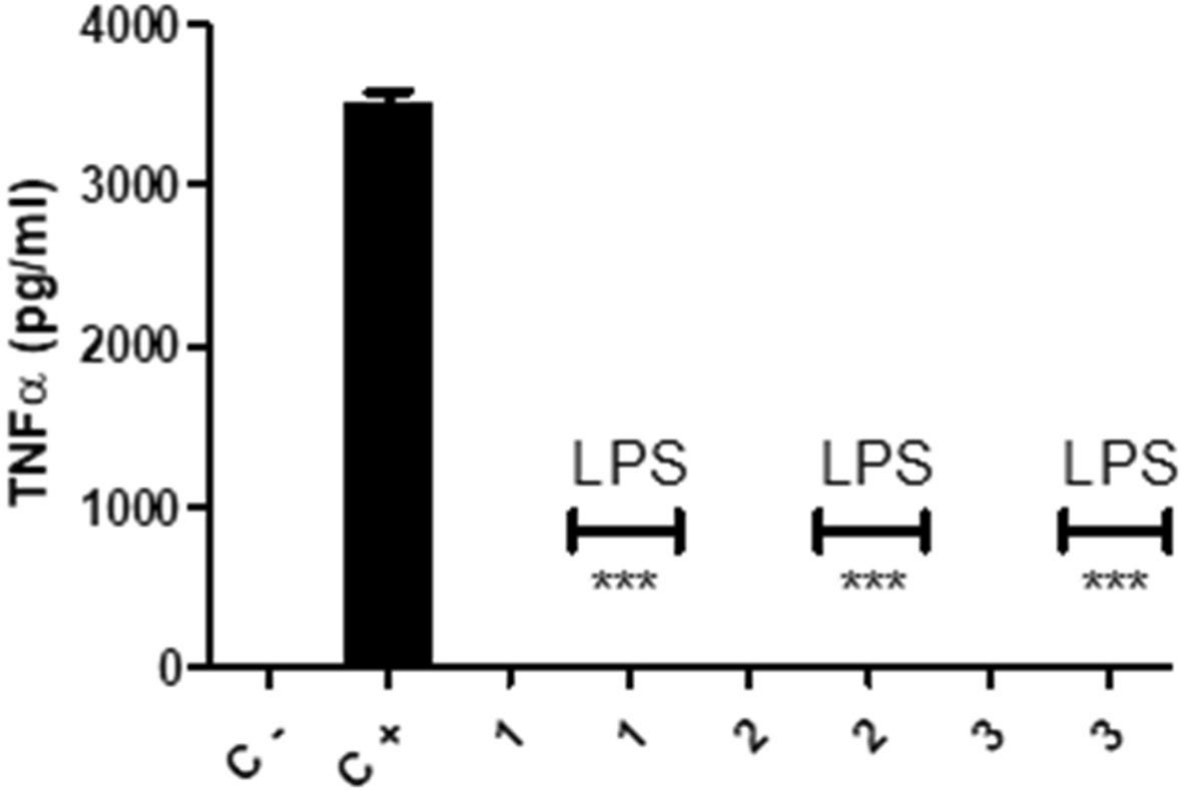
TNF ELISA results for the supernatants of the macrophages incubated 24 h on the PCL1, 2 and 3 fiber sheets. These sheets were covering the bottom of the 24 well plates and the cell concentration was 1 × 10^6^ cells/mL. The cells were rested 12 h on the sheets later on they were either stimulated with 1 µg/mL LPS or incubated without LPS. Control negative and positive wells did not have any PCL fiber sheet covering the bottom of the wells. In positive control well 11 µg/mL LPS was used for macrophage stimulation. T test was done for the statistics, *p* < 0.0001 N = 3.

**Figure 5:**
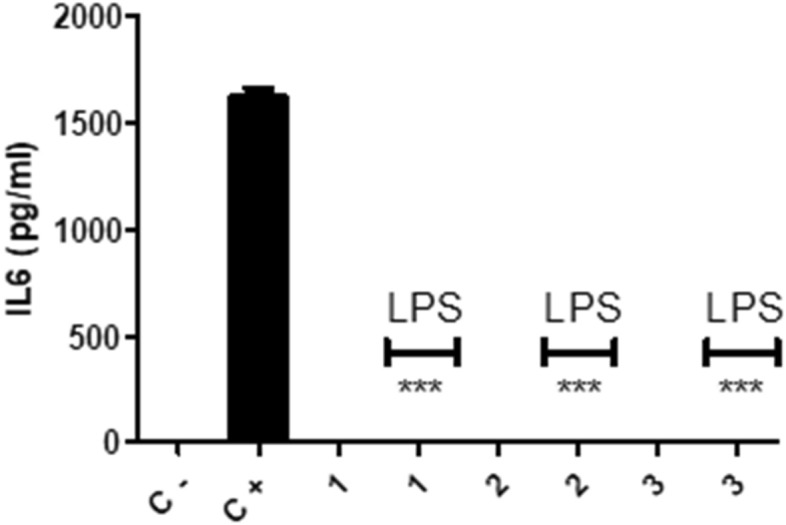
IL6 ELISA results for the supernatants of the macrophages incubated 24 h on the PCL1, 2 and 3 fiber sheets. These sheets were covering the bottom of the 24 well plates and the cell concentration was 1 × 10^6^ cells/mL. The cells were rested 12 h on the sheets later on they were either stimulated with 1 µg/mL LPS or incubated without LPS. Control negative and positive wells did not have any PCL fiber sheet covering the bottom of the wells. In positive control well 11 µg/mL LPS was used for macrophage stimulation. T test was done for the statistics, *p* < 0.0001 N = 3.

In order to test either immunostimulatory or immunomodulatory functions of these nanofibers the macrophages were first seeded on 24 well plates that whose bottom were fully covered with PCL1, PCL2 and PCL3 nanofiber sheets separately. For the induction of their inflammatory reaction 1 µg/mL LPS was added into the wells. To test the immunostimulatory function of the nanofiber sheets the incubated macrophages were not activated by LPS. TNF and IL6 inflammatory cytokine levels were measured after 24 h of incubation. These cytokines are hallmark pro-inflammatory cytokines of macrophages. In positive control wells where the bottom of the wells was not covered with nanofiber sheets there was substantial production of both cytokines (Figs. 4 and 5). Nanofiber sheets lacked immunostimulatory activity since in the absence of LPS they did not stimulate macrophages to produce pro-inflammatory TNF and IL6 cytokines. Compared to positive control groups, macrophages had complete knockout of TNF and IL6 pro-inflammatory cytokine secretion upon their activation with LPS when they were seeded on PCL nanofibers. Having a different diameter for each nanofiber type did not make any difference when they were in sheet form (Figs. 4 and 5). The results were similar between the groups. Most probably the reason behind it was macrophage behavior in the fibrous mats (Fig. 2). Macrophages were dispersed around the mats and this resulted in less contact between the macrophages. These cells need to contact with each other to exert their full inflammatory potential^53^. Previous studies with nanofibers also indicate that mammalian cells can attach to them efficiently and this attachment, in some cases, increases the rate of the cell proliferation^12–14^.

## Discussion

PCL is widely used in the production of scaffolds in different forms developed for use in tissue engineering applications with its proven biocompatibility. Among them, PCL nanofibrous scaffolds fabricated by electrospinning are of great interest—due to their easy production, high surface area and good mechanical stability. Besides all these features, it is still not clear how these scaffolds will affect the immune system and macrophage behavior during and after implantation. In addressing this situation, we aimed to determine the immunomodulatory activity of PCL nanofibers, which are frequently used in tissue engineering field, and to demonstrate whether the changing fiber diameter affects the immune response. With this aim, PCL nanofibrous scaffolds at different fiber diameters were produced via electrospinning method by varying the operation parameters. The RAW 264.7 mammalian macrophages were used in order to test the immunostimulatory and immunomodulatory functions of these fibrous scaffolds. Our results show that macrophages can easily attach and disperse into the surface of the fabricated scaffolds. In addition, all samples showed a decrease in M1 phenotype’s inflammatory cytokines TNF and IL6. However, the change in diameter did not have major effects on these results. This may be the result of the dispersion of macrophages all around the fibrous mats, causing less contact between nanofibers and macrophages.

In our study, we observed that macrophage attachment and dispersion in the nanofibers eliminated their pro-inflammatory activities. Having different diameters did not alter the outcome in this study. Previous studies also suggest that having modified forms of PCL changes the inflammatory behavior of macrophages and polarizes them toward M2 phenotype rather than M1 phenotype^16^. Our results are in line with the previous findings in terms of decrease in M1 phenotype’s inflammatory cytokines TNF and IL6^16^.

Our results support possible utilization of the nanofibers in tissue engineering, organ transfer or implantation where the suppression of inflammatory responses is crucial to prevent toxicity, organ or implant rejection^2–5^. PCL nanofibers most probably function by caging macrophages on their mats and preventing their contact activation during LPS stimulation. This would prevent excessive inflammation during organ transfer, tissue engineering and implant positioning in vivo^2–5^. The diameter differences between the PCL nanofibers that we utilized in our study did not change the outcome of the macrophage inflammatory responses. All of these PCL nanofibers can be potentially utilized as materials during tissue, organ and implant transfer to the body to cage macrophages on their surfaces and prevent their inflammatory responses.

There are studies suggesting that PCL nanofibers can direct migration and differentiation of different types of mammalian cells when these fibers were coated with tissue or polarization related junction proteins and integrins^54^. It would also be interesting to decipher the interaction between the integrin molecules of macrophages and PCL nanofibers for their attachment and how this might also alter their activation pattern.

In our future studies we will be deciphering their effects on different immune system cells, their molecular mechanism of action with different integrin signaling systems and in vivo activities. In addition, instead of evaluating the fiber diameter alone, it has become clear that a new optimization study should be carried out by changing the alignment of fibers, fiber density, membrane thickness and porosity between fibers, supported by in vitro and in vivo experiments.

## Data Availability

The data will be made available by the corresponding authors upon reasonable request.
